# Evaluation of a teacher training program to enhance executive functions in preschool children

**DOI:** 10.1371/journal.pone.0197454

**Published:** 2018-05-24

**Authors:** Laura M. Walk, Wiebke F. Evers, Sonja Quante, Katrin Hille

**Affiliations:** 1 ZNL TransferCenter for Neuroscience and Learning, Ulm University, Ulm, Germany; 2 Department of Psychology, University of Heidelberg, Heidelberg, Germany; Georgetown University, UNITED STATES

## Abstract

**Background:**

Executive functions (EFs) play a critical role in cognitive and social development. During preschool years, children show not only rapid improvement in their EFs, but also appear sensitive to developmentally appropriate interventions.

**Aim:**

EMIL is a training program for German preschool teachers that was developed and implemented to improve the EFs of preschoolers. The aim of the present study was to evaluate its effects on the EFs of children between three and six years old.

**Method:**

The teacher training (eight sessions, 28.5 hours) was implemented in four preschools. The EFs of children of the intervention group (*n* = 72, 32 girls, *M*_age_ = 48 months) and the control group of four other matched preschools (*n* = 61, 27 girls, *M*_age_ = 48 months) were tested before, during, and after the intervention using different measures assessing working memory, inhibitory control, and cognitive flexibility.

**Results:**

The intervention group showed significant gains on three out of seven EF tests (behavioral inhibition, visual-spatial working memory, and combined EFs) compared to the control group. Post hoc analyses for children with low initial EFs scores revealed that participation in the intervention led to significant gains in inhibitory control, visual-spatial working memory, and phonological working memory as well as a marginally significant difference for combined EFs. However, effect sizes were rather small.

**Conclusion:**

The results suggest that teacher training can lead to significant improvements in preschooler’s EFs. Although preliminary, the results could contribute to the discussion on how teacher training can facilitate the improvement of EFs in preschool children.

## Introduction

Young children often act ‘in the heat of the moment’. They act impulsively, can be easily distracted, have trouble waiting or sitting still for a prolonged period, and show little persistence in what they do [1]. These skills of cognitive control and behavioral regulation are often referred to as executive functions (EFs). EFs are top-down mental processes that enable goal-directed action as well as adaptive responses to novel or challenging situations and unfamiliar tasks when acting automatically would be inadequate or impossible [2, 3]. EFs make it possible to concentrate and pay attention, take the time to think before acting, control feelings, and resist temptations [4]. Thus, they are important for higher cognitive functions such as planning, judgement, decision making, structuring and execution of tasks, and for the detection and correction of errors [5–7]. EFs encompass three separable but related components: working memory, inhibitory control, and cognitive flexibility [8]. Working memory is the ability to maintain, update, and monitor information and to mentally work with or manipulate this information. Hence, working memory is important to plan and act in a goal-directed manner and stay on task. Inhibitory control or inhibition describes the ability to resist a first inclination and think before acting. It is needed to control impulses and ignore distracting stimuli in order to stay focused. Cognitive flexibility is the ability to shift between tasks or mental sets and adjust to changed demands or precedencies. It is also important to take on a new perspective and to switch between perspectives [9, 10]. These cognitive skills form the basis for intentional self-controlled behavior and are of great importance in doing well at school, work, and in mastering the demands of daily life [11].

Over the years, numerous studies have shown that EFs are associated with the cognitive and social-emotional development of children and predict many life outcomes: EFs show significant correlations with academic achievement and school success [12–14]. Better EFs are related to proficiency in mathematics [15–18], reading and literacy [15, 19, 20], and the ability of verbal and nonverbal reasoning [21]. Studies have shown that the EFs of preschoolers predict their school readiness and later academic performance [12, 18, 22]. In addition, EFs are correlated with social competence, socially appropriate behavior [23–25], and emotion regulation [26].

EFs emerge in early childhood and continue to develop over childhood and adolescence until early adulthood [27]. This long period of development is related to the maturation of the prefrontal cortex where the neural correlates are found [28]. This area undergoes one of the longest periods of development; only in young adulthood this region is fully developed [29, 30]. Since it has been demonstrated that EFs improve rapidly through the preschool and elementary school years [29, 31] and the brain has greater neural and behavioral plasticity during early childhood [32], those time periods bear great potential for the enhancement of EFs [33, 34].

Studies have shown that different games, activities and exercises promote the EFs of preschool children, for example circle games, pretend-play, and physical activity [35–37]. Moreover, specially developed curricula for preschool and primary schools such as Tools of the Mind and Promoting Alternative Thinking Strategies (PATHS) aim to strengthen children’s EFs [10, 38, 39]. Those curricula are based on a teacher training that implement the promotion of EFs into both daily routine and special lessons. It should be noted, however, that the evaluation studies of these curricula come to inconclusive results [10, 40].

One reason may be that various factors that are associated with EFs such as gender, parents’ education, socio-economic status, and employment level [41–44] and have shown to mediate the impact of cognitive training programs on the improvement of children’s EFs [38, 45], are not always sufficiently controlled for.

Several principles have to be taken into acount when attempting to enhance the development of EFs in young children [46]:

1. EFs must be continually challenged to see improvements. For that, it is crucial to create individual curricula and programs to enable an appropriate challenge for each child.
2. There is a greater chance to get transfer effects on other cognitive, social, and behavioral competencies if the training program of EFs is more global. Programs should address many EF components and self-regulatory skills, such as in fully integrated curricula [47, 48].
3. Another key element is repetition. EFs benefit the most if they are addressed again and again. Training throughout the day and every day produces more improvements than lessons only once or twice per week.
4. Intervention studies showed that preschool programs and curricula do not need expensive equipment to support EFs [49]. Teachers need basic knowledge, further education, and professional support on how to promote children’s EFs.
5. In order to achieve the best results in improving EFs it is important to bear in mind children’s emotional and social development. EF activities should bring joy and confidence to make sure children are willing to devote time to them.
6. Because of the process of development and the rapid improvement of EFs in the preschool years, interventions should start as early as possible [14].

Despite this knowledge and the growing interest in promoting EFs in children, there is no early childhood education program to enhance EFs in Germany.

The newly developed EMIL intervention aims at strengthening preschoolers EFs through teacher training [50, 51].Public preschools provide optimal access to the majority of children (about 80% of all three- to six-year-olds attend preschool). Implementing an education program in the preschool setting could equip children with competencies to be successful in school and life, and possibly reduce the achievement gap [52].EMIL was developed on the basis of research findings, in line with the workings of German preschools, and with the help of preschool teachers. All before mentioned principles were taken into account in the development: EMIL focuses on individual promotion in a social context. All activities are integrated into everyday life. As a result, a continuous promotion of EFs takes place. The implemented activities and exercises are playful and appropriate to the current needs and skills of the children.

The aim of the present study is to evaluate whether participation in the EMIL teacher training leads to gains in the EFs of preschool children.

## Methods

### Ethics statement

The study was approved by the local ethical committee of the Ulm University. All research procedures and participant recruitment materials were reviewed and approved. Parents of participating children were informed about the procedures of the study before giving their written consent. Participating children were given verbal information about the procedure.

### Participants

Eight preschools took part in the study. They all belonged to the same school district and worked according to the same pedagogical concept called ‘infans’ [53]. In total, 67 preschool teachers (64 female, 3 male) and 133 children (74 male, 59 female) participated in the study. Children were recruited through letters distributed by the preschools. Information events were organized at each of the eight preschools for the parents to ask questions. At the time of first testing, children’s age ranged from 30 to 60 months (*M*_age_ = 48 months, *SD* = 7.65 months). The intervention group (four preschools with 33 teachers) consisted of 72 children (32 girls, 40 boys, *M*_age_ = 48 months, *SD* = 7.8 months), the control group (the other four preschools with 34 teachers) of 61 children (27 girls, 34 boys, *M*_age_ = 48 months, *SD* = 7.5 months).

### Design

The preschools that took part in the study had open classrooms. In 2015, the open classroom concept was implemented in more than 13% of all preschools in Germany, making it a well-established concept of preschool care [54]. In open classrooms children are not assigned to a particular teacher or class. The children are free to move from one room to another and work with different teachers and children according to their wishes and needs. Subsequently, in the present study it was not possible to assign groups at classroom level but only at preschool level. The individual preschools varied in size as well as in the age distribution and socio-economic status (SES) of the children. To control for these differences, preschools were paired based on the following criteria: number of children in preschool, sex and age distribution, proportion with immigration background, and SES (estimated by the preschool principal). One preschool of each pair was randomly assigned to the intervention. The other became the “practice as usual” control group.

**Fig 1:**
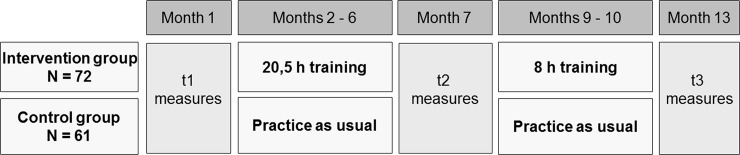
Study design.

Fig 1 shows the study design. In order to gain insight in the children’s development of EFs, they were tested at three different times over the course of one year (before the intervention, seven months into the intervention, and two months after). Over a period of nine months, the 33 preschool teachers of the EMIL intervention group received eight training sessions (total number of hours: 28.5 h).

### EMIL—An intervention program to enhance EF skills

EMIL is an intervention program designed to enhance EF skills in children. It consists of a teacher training program and its implementation in preschools [51, 55]. Research findings and the experiences of preschool teachers both contributed to its development. The training program consisted of eight sessions. Five training sessions were carried out with all preschool teachers of the four intervention preschools together. The other three sessions took place with each preschool team individually at their preschool.

The sessions involved presentations on the definition, meaning, development, and training of EFs. The preschool teachers learned about the cognitive and social-emotional development of children, the development of EFs and their importance for social and academic skills. They also learned different ways to enhance EFs in the preschool setting. The teachers reflected on these aspects and discussed ideas on how they can modify demands on EFs and enhance EFs in the daily routines of their own preschool. Different methods and activities that were proven to enhance EFs (e.g., physical and mindfulness activities, role-play) were introduced and practiced. The teachers received materials to give them ideas on how to transfer what was learned into practice at their preschool.

EMIL covers four aspects that can be addressed in all German preschools regardless of their pedagogical concept: 1.) attitude (teacher’s mindset and perception of the child); 2.) interaction & communication (e.g., talking with children and asking questions to activate and involve the child); 3.) structures (set times, interior design/provided materials, rules/rituals, and group structure); and 4.) activities (e.g., games, physical activity, pretend-play, relaxation and mindfulness techniques).

The EMIL training program was delivered to complete teams of preschool teachers. That was important because in Germany many preschools have an open classroom concept, requiring a common understanding and approval of daily routines by the team. By having the whole team taking part in the training program, they were able to work together on solutions for their preschool.

While the teacher training program has a predetermined structure with predefined contents, how teachers incorporate these ideas in their daily work was not imposed on them. EMIL intended to enable preschool teachers to adjust their work in a way that enhances children’s EFs purposefully but en passant. In this regard, EMIL is neither a preschool concept nor an add-on but rather an add-in that is supposed to be easily integrated into various preschool environments.

The teachers were expected to identify situations and opportunities in their preschool based on examples discussed during the training sessions and to support children individually, depending on the situation. In this way, EMIL is meant to be integrated into various preschool curricula and to be adapted to the needs of differing preschools, children and organizational structures. This transfer to differing settings was supported by three aspects of the teacher training:

1. Teachers acquired an ‘EF-lens’. That means they learnt to see preschool situations in terms of EFs considering typical situations like lunch, playing board games, and doing sports as opportunities to enhance children’s EFs.
2. Teachers worked on the issues of their preschool. Different preschools face different challenges. During the training sessions, the teachers were encouraged to apply the new knowledge to their own setting and set realistic goals for improvements.
3. Teachers were encouraged to see the resources of their preschool and the strengths of their children. Rather than focusing on weaknesses, the trainer helped the teachers focus on enhancing those strengths.

In the course of the teacher training, one preschool team for example decided to improve EF skills and self-regulated behavior by changing the room layout. They created retreat opportunities for the youngest and provided more space for playing with building blocks for the older children. With this modification children could easier meet their own needs and regulate their own emotions. As a result, there were fewer situations of excessive EF demands and conflicts among the children. Another preschool team provided more time for physical activities to improve EFs and played more games like “Simon says” for impulse control. They incorporated in their daily routines good old familiar games as well as new games to strengthen EFs in children.

Various changes of this kind have been implemented by all four preschools—some of them were more extensive, others rather small, but all were in line with the individual situation and the aim of the EMIL program.

### Testing procedure

EFs were assessed using seven different tasks appropriate for the age group. The tasks were administered by the test administrators in two individual sessions, each lasting 30 minutes, in a quiet room at the preschools. All children received the same test battery. All tasks were presented in a fixed order for all children [56]: Tower, Block Recall, and Head-Toes-Knees-Shoulders were part of the first session. Hearts & Flowers, Day & Night, and Digit Span forward and backward were part of the second session.

### Measures

#### Behavioral inhibition

In the *Tower* task [57], the child takes turns with the test administrator to build a tall tower out of wooden building blocks. After a brief demonstration of turn-taking, the test administrator begins building the tower. Afterwards, the test administrator does not take a turn unless the child communicates that it is her turn, e.g., verbally or by handing her a block. Different from the original task that uses 20 blocks, each child was presented with 15 building blocks. Children’s mistakes (placing a block when it was not their turn) were counted. The sum of the two trials was used in analyses (max. 26 mistakes).

#### Inhibitory control

In the *Day & Night* task [9, 58], the child is asked to react to 16 pictures of either a sun or a moon appearing in random order on the computer screen. The child is told to say”sun” whenever the moon appears on the screen and say “moon” when the sun appears. After four practice trials, no feedback was given to the child during the 16 trials. The child was awarded two points for every correct answer, one if the child corrected themselves and zero points if the answer was wrong (max. 32 points).

#### Behavioral self-regulation

The *Head-Toes-Knees-Shoulders* task (HTKS) [42, 59] requires children to respond to commands first naturally and then in the “opposite” way. There are rules the child has to remember to respond correctly to behavioral commands. In the first of the three parts, the child was told to touch his/her head when the command “Touch your toes” was given, and to touch his/her toes when the command “Touch your head” was given. After two questions to check understanding, children were given four practice trials and the instructions were repeated up to three times during the practice trials. Then ten test commands were given verbally in random but fixed order, without feedback. In the second part, two additional commands where introduced. When asked to “Touch your shoulders”, the child had to touch his/her knees. When asked to “Touch your knees”, the child had to touch his/her shoulders. In the third part, all commands changed. Now, when asked to “Touch your head”, the child had to touch his/her knees and vice versa. When asked to “Touch your shoulders”, the child had to touch his/her toes and vice versa. During the testing portion, the test administrator stated the behavioral commands without modeling any actions. Children received two points for a correct response, one point for a self-correct, defined as making any discernable motion (ranging from slight to complete) toward the incorrect response but ending with the correct response. Zero points were given for an incorrect response. If children received at least four points on the 10 items, the next rule was added. For the three parts with 30 trials the total score was 60 points.

#### Visual-spatial working memory

In the *Block Recall* task [60] the child is asked to remember a sequence that the test administrator shows. The child is presented with nine wooden cubes located randomly on a board. The test administrator taps a sequence of blocks with a stick. The child’s task is to tap with his/her own stick the same blocks in the same order. The test administrator begins with tapping one block. The length of the sequence is increased by one additional block when two sequences of the same length are correctly repeated by the child. The child is awarded one point for each correct repetition (max. 21 points).

#### Phonological working memory

The *Digit Span forward* and the *Digit Span backward* task [61] were used to assess phonological working memory. The digit recall test requires the child to repeat a sequence of digits in correct serial order. Lists of the digits 1 to 9 are read out aloud by the test administrator at the rate of one digit per second. Following a short practice session with two trials, the test administrator reads out a maximum of four lists of each length. List length is increased by one if the child recalls three lists at that length correctly. Testing continued until the child recalled two lists of one length incorrectly. The number of lists correctly recalled is scored (max. 12 points). The backward digit recall test is identical to the forward digit recall test in all respects except that it requires the child to recall the sequence of spoken digits in reverse order. Practice trials were given in order to ensure that the child understands the concept of “reverse”. The number of lists repeated correctly was scored (max. 6 points).

#### Combined EFs (working memory, inhibitory control, and cognitive flexibility)

The *Hearts & Flowers* task [10, 62] requires the child to react correctly and as fast as possible to a stimulus (red heart or blue flower) presented on a computer screen (right or left side). Depending on the stimulus and the side of the screen that it appears on, the child has to press one of two buttons as quickly as possible. *Hearts & Flowers* contains three conditions with 10 practice and 40 testing trials each. The congruent condition places minimal demands on EFs. The child is instructed “to press the button on the same side as the heart”. Therefore, the child has to remember the rule and apply it as quickly and correctly as possible. The incongruent condition demands, in addition to working memory, inhibitory control. It requires the children remember the rule “to press on the side opposite the flower” and inhibit the tendency to follow the rule of the congruent condition. The mixed condition demands all three core EF skills (inhibitory control, working memory, and cognitive flexibility). Red hearts and blue flowers appear in a random order on either the right or the left side of the screen. Children need to apply the rules of the congruent and the incongruent condition flexibly. In all three conditions, children were given seven seconds to respond. If they responded too fast (in less than 0.4 seconds) the response was not taken into account. If they responded too slow, it was counted as an error. Children received one point for every correct response (max. 120 points).

#### Family demographics

Primary caregivers were asked to complete a short background questionnaire on the family including citizenships, languages spoken in the home, and family income.

#### Teacher satisfaction

After every EMIL training session, a short questionnaire was handed to all participating teachers. The teachers were asked to rate their level of satisfaction on a scale from 1 to 6 (1 = ‘very satisfied’ to 6 = ‘not satisfied at all’) concerning the following aspects of the EMIL training: learning content, instruction methods, relevance for teaching practice, learning outcomes, and collaboration with the EMIL instructors.

### Missing data

Not all children were able to take part in all testing sessions due to extraneous circumstances (e.g., being sick or on vacation). At the t2 assessment, one child of the control group did not take part in the second of the two testing session due to prolonged absence from school. At t3, three children of the intervention group were not present for the first testing session and 3 children (one of the control and two of the intervention group) were absent during the second testing session. Also, not all children that were present chose to participate in every test presented to them in the testing session. In other cases, their performance on the tests could not be analyzed due to obvious problems understanding the instructions. The test with the highest refusal or abortion rate was the Hearts & Flowers which was the longest and most monotonous task of the session.

The return rate for the caregiver questionnaire was 97% in the intervention group and 89% in the control group. However, some parents did not answer all questions in the questionnaire. Response rates were especially low for family income with data missing for 23 out of the 124 children whose parents filled in the questionnaire.

Still, data were assumed to be missing completely at random (MCAR). Little’s MCAR test [63] was performed for all dependent and independent variables used in the following analyses. Little’s MCAR did not reach significance, suggesting that data were indeed missing completely at random (χ^2^ (638, N = 133) = 628.03, p = .603). Missing data were not imputed.

### Statistical analyses

IBM SPSS Statistics, version 22.0 for Windows [64] was used for the statistical analyses. Chi-Square tests were conducted to check for differences in children’s background variables (e.g., gender, age) and family demographics (e.g., educational and occupational level of parents) between the intervention and the control group. Pearson’s correlation coefficients were calculated between children’s background and family variables and EF measures.

Univariate Analyses of Covariance (ANCOVAs) were conducted to assess group differences in performance gains on EF measures. ANCOVAs were preferred over repeated measures Analyses of Variance because they provide a more accurate estimate of the effect of the intervention as they control for pre-existing differences [65]. Group (intervention vs. control) served as the between-subject factor. Age, maternal and paternal educational and occupational level, and t1 pre-test score for each individual test were entered as covariates. Dependent variables were EF scores at t2 and t3. An estimate of the effect size, partial eta squared (η^2^), was calculated for each dependent variable.

## Results

Table 1 presents the response rate for each construct, descriptive statistics, and Chi-Square test results for the intervention and the control group. The children belonged mostly to well-educated families. Almost 90% of parents were German citizens. About 8% of the children did not speak German as their first language. The sample can be considered representative for the population from which it was drawn. Chi-Square tests showed no significant differences between the two groups in any of the demographic variables. The matching process appeared effective in creating comparable groups prior to intervention. However, there are some tendencies observed for both parental educational and occupational background.

**Table 1:** Descriptive statistics for child background and family variables.

Demographic variables	Total		Intervention Group		Control Group		Differences between groups	
	%	N	%	N	%	N	*X*^*2*^	*p*
**Sex**								
male	55.5	74	55.6	40	55.7	34	.00	.98
female	44.5	59	44.4	32	44.3	27		
**First language German**								
no	8.9	11	11.4	8	5.7	3	1.23	.27
yes	91.1	112	88.6	62	94.3	50		
**Mother German citizen**								
no	13.0	16	11.6	8	14.8	8	.28	.60
yes	87.0	107	88.4	61	85.2	46		
**Father German citizen**								
no	14.9	18	14.5	10	15.4	8	.02	.89
yes	85.1	103	85.5	59	84.6	44		
**Time in preschool per day**								
3–4 hours	24.6	30	30.4	21	17.0	9	3.79	.15
5–6 hours	31.1	38	31.9	22	30.2	16		
7–8 hours	44.3	54	37.7	26	52.8	28		
**Monthly family income**								
less than 1500 €	11.9	12	8.6	5	16.3	7	1.92	.75
between 1500 and 2000 €	16.8	17	17.2	10	16.3	7		
between 2000 and 3000 €	23.8	24	25.9	15	20.9	9		
between 3000 and 4000 €	18.8	19	17.2	10	20.9	9		
4000 € and more	28.7	29	31.0	18	25.6	11		
**Mother’s educational level**								
9 years or les	14.9	18	9.0	6	22.2	12	4.19	.12
10 years	35.5	43	37.3	25	33.3	18		
12 years or more	49.6	60	53.7	36	44.4	24		
**Father’s educational level**								
9 years or less	17.2	20	12.3	8	23.5	12	3.00	.22
10 years	28.4	33	27.7	18	29.4	15		
12 years or more	54.3	63	60.0	39	47.1	24		
**Mother’s occupational level**								
No training	8.3	10	4.5	3	13.0	7	4.09	.25
Vocational training	52.9	64	52.2	35	53.7	29		
Academic degree	29.8	36	31.3	21	27.8	15		
Doctoral degree	9.1	11	11.9	8	5.6	3		
**Father’s occupational level**								
No training	10.8	13	10.3	7	11.5	6	2.07	.56
Vocational training	43.3	52	38.2	26	50.0	26		
Academic degree	30.0	36	33.8.8	23	25.0	13		
Doctoral degree	15.8	19	17.6	12	13.5	7		

Descriptive statistics (i.e., means, standard deviations, possible score ranges) for the EF tasks for all three assessments are shown in Table 2. The percentage of missing values per EF task ranged between 0 and 5%, with the exception of up to 9% in the second and third condition of the HTKS and the Hearts & Flowers task, which is mostly attributable to the duration of both tasks. Ceiling effects were noted for the Tower at t3. Floor effects were found for Digit Span backwards at t1 and t2.

**Table 2:** Means and standard deviations of outcome variables by condition and measurement time.

Measure (Range)	Group	t1			t2			t3		
		N	Mean	SD	N	Mean	SD	N	Mean	SD
**Tower** **(0–26)**	Intervention	72	5.89	7.12	71	1.83	3.72	69	1.57	4.37
	Control	59	4.29	7.45	60	4.60	7.63	59	2.98	6.61
**Day & Night** **(0–32)**	Intervention	72	20.01	10.11	72	24.35	9.05	70	26.96	6.81
	Control	60	22.23	7.29	58	24.31	9.15	58	26.71	8.17
**HTKS** **(0–60)**	Intervention	70	15.01	16.02	72	24.46	17.49	69	31.59	17.96
	Control	57	16.95	16.58	56	24.96	17.45	57	33.11	18.49
**Block Recall** **(0–21)**	Intervention	72	5.64	3.07	72	7.49	3.52	69	9.43	2.70
	Control	60	5.63	3.34	60	7.30	3.14	60	7.72	3.79
**Digit Span forward** **(0–12)**	Intervention	72	5.04	1.96	72	5.96	1.61	70	6.23	1.69
	Control	59	5.15	1.68	58	5.45	1.91	58	5.81	1.58
**Digit Span backward** **(0–6)**	Intervention	72	.43	1.02	69	1.07	1.31	68	1.71	1.60
	Control	56	.61	1.07	53	1.26	1.32	57	1.93	1.60
**H&F** **congruent (0–40)**	Intervention	71	24.89	9.62	71	30.14	10.35	69	32.80	9.10
	Control	59	26.34	9.73	57	27.49	11.57	57	29.09	9.91
**H&F** **incongruent** **(0–40)**	Intervention	70	21.00	10.54	71	26.01	11.97	68	29.93	10.79
	Control	58	23.03	10.21	55	26.02	11.28	57	27.70	10.77
**H&F** **mix** **(0–40)**	Intervention	68	21.21	7.65	68	25.68	9.95	68	28.29	9.90
	Control	57	23.81	9.04	55	25.65	9.33	56	27.50	9.75

HTKS = Head Toes Knees Shoulders Task, H&F = Hearts & Flowers.

Table 3 shows the correlations among EF test scores at pre-assessment as well as with children’s background variables. All EF measures show significant correlations with one another, except for the HTKS and Digit Span forward (p = .065). The highest correlations (r = .660 and r = .642, both ps < .001) were found among the three subtests of the Hearts & Flowers task. Significant correlations with age were found for all EF measures, which was not surprising given the age range of the participants. When controlling for age, significant correlations were found for time spend at preschool and inhibitory control (Day & Night), maternal educational level and working memory (Digit Span forward), both paternal educational and maternal occupational level and combined EFs (Hearts & Flowers mix) as well as for paternal educational level and combined EFs (Hearts & Flowers incongruent). Significant correlations were also found between family income and both working memory (Digit Span forward) and combined EFs (Hearts & Flowers congruent).

**Table 3:** Correlations among EF test scores at pre-assessment (t1) and partial correlations with children’s background variables controlling for age.

		1	2	3	4	5	6	7	8	9
**EF test scores at pre-assessment**										
1	Tower	---								
2	Day & Night	-.358**	---							
3	HTKS	-.377**	.331**	---						
4	Block Recall	-.383**	.319**	.439**	---					
5	Digit Span forward	-.317**	.352**	.359**	.425**	---				
6	Digit Span backward	-.235**	.163	.467**	.386**	.342**	---			
7	H&F congruent	-.399**	.314**	.574**	.408**	.458**	.371**	---		
8	H&F inconcrunet	-.294**	.191*	.454**	.382**	.275**	.338**	.288**		
9	H&F mix	-.353**	.291**	.546**	.430**	.316**	.476**	.660**	.642**	
**Children’s background variables**										
Age		-.406**	.375**	.517**	.521**	.484**	.377**	.443**	.422**	.489**
**Partial correlations controlling forage**										
Time in preschool		-.063	.265*	.020	-.094	-.018	.021	.081	.008	-.018
Maternal educational level		-.186	.074	.086	.104	.228*	.069	.141	.106	.134
Paternal educational level		-.102	.115	.215	.021	.077	.060	.165	.235*	.283*
Maternal occupational level		-.089	.102	.159	.194	.236*	.103	.233*	.087	.232*
Paternal occupational level		-.167	.070	.031	.070	.025	-.026	.187	-.003	.200
Family Income		**-**.199	.039	.178	.156	.247*	.211	.237*	.162	.200

HTKS = Head-Toes-Knees-Shoulders task; H&F = Hearts & Flowers.

*p < .05

**p < .01.

To test the efficacy of the teacher training, a between-group comparison (intervention vs. control group) using analyses of covariance was conducted. ANCOVAs were conducted once to compare performances between the intervention and the control group at t2 and again to compare performances at t3. The selection of covariates (age and educational and occupational level of both parents in addition to performance at t1) was supported by the results of the Chi-square tests and the correlational analyses. The results of the ANCOVAs are presented in Table 4. Note that the due to missing data in the caregiver questionnaire the entering of covariates lowered the sample sizes in both the intervention and the control group. At t2, significant intervention effects were found only for the Tower [F(1, 105) = 16.73, p < 0.01, partial eta^2^ = .14]. Performance on Tower at t2 was associated with performance on Tower at t1. Hearts & Flowers congruent [F(1, 100) = 3.19, p = 0.07, partial eta^2^ = .03] showed marginally significant differences between the two groups at t2. Performance on Hearts & Flowers congruent at t2 was influenced by age and performance at t1.

**Table 4:** Comparison of the performances of the intervention and control group on the EF tasks at t2 and t3: means, standard deviations, and results of between-group ANCOVAs.

Measure	Group	t2							t3						
		N	M	SD	*df*	F	*p*	eta^2^	N	M	SD	*df*	F	*p*	eta^2^
**Tower**	IG	63	1.81	3.84	**1, 105**	**16.73**	**.00**	**.14**	61	1.70	4.62	1, 103	1.86	.18	.02
	CG	50	4.64	7.65					50	2.94	6.75				
**Day & Night**	IG	64	24.06	9.27	1, 104	0.12	.91	.00	62	27.00	6.65	1, 102	0.05	.83	.00
	CG	48	24.19	9.34					48	26.96	8.26				
**HTKS**	IG	62	24.89	17.75	1, 100	0.23	.63	.00	59	31.75	17.49	1, 98	0.31	.58	.00
	CG	46	25.52	17.87					47	33.68	18.75				
**Block Recall**	IG	64	7.44	3.64	1, 106	0.10	.75	.00	61	9.26	2.75	**1, 103**	**9.60**	**.00**	**.09**
	CG	50	7.26	3.06					50	7.84	3.93				
**Digit Span forward**	IG	64	5.91	1.64	1, 103	2.57	.11	.02	62	6.16	1.74	1, 102	0.92	.34	.01
	CG	47	5.62	1.84					48	5.98	1.62				
**Digit Span backward**	IG	62	1.00	1.31	1, 95	0.63	.43	.01	61	1.61	1.54	1, 98	2.70	.10	.03
	CG	41	1.41	1.34					45	2.07	1.62				
**H&F congruent**	IG	62	30.15	10.51	**1, 100**	**3.19**	**.08**	**.03**	60	32.53	9.17	**1, 99**	**4.36**	**.04**	**.04**
	CG	46	27.91	11.70					47	29.04	10.56				
**H&F** **incongruent**	IG	61	26.54	12.06	1, 97	0.22	.64	.00	59	29.05	11.09	1, 98	0.14	.71	.00
	CG	44	27.59	9.89					47	28.55	10.11				
**H&F** **mix**	IG	58	25.66	10.33	1, 93	0.05	.82	.00	57	28.60	10.06	1, 94	0.04	.85	.00
	CG	43	26.79	9.04					45	28.09	9.46				

IG = Intervention group; CG = Control group.

The following variables served as covariates: performance at t1, age, maternal and paternal educational level, and maternal and paternal occupational level. Significant differences and near-significant trends are highlighted.

At t3, significant differences between the intervention and the control group were found for the Block Recall [F(1, 100) = 9.41, p < 0.01, partial eta^2^ = .09]. Both age and performance at t1 affected performance at t3. Additionally, a significant differences were found for Hearts & Flowers congruent [F(1, 99) = 4.36, p = 0.04, partial eta^2^ = .04]. Performance was influenced by age and performance at t1. No significant intervention effects were found in the other EF tests: Day & Night, HTKS, Digit Span forward and backward, and Hearts & Flowers incongruent and mix.

Previous studies have found effects for children with lower initial levels of EFs even when no effects became evident for the overall sample [38, 49, 66]. To test this assumption, post hoc analyses with the same covariates were carried out for children scoring at or below the 50th percentile. Significant differences in favor of the intervention group were found for the following tests: Tower and Digit Span forward at t2 and Block Recall at t3. Of the children scoring initially low on the Tower (1 or more mistakes), 43 belonged to the intervention and 25 to the control group. At t2, the children of the intervention group obtained an average score of 2.95 (SD = 4.62) and the children of the control group an average score of 8.30 (SD = 8.50). As lower scores indicate better performance, the intervention group outperformed the control group at t2 [F(1, 50) = 11.14, p < 0.01, partial eta^2^ = .18]. Of the children scoring low on the Block Recall at t1 (5 points or less), 36 belonged to the intervention group and 33 to the control group. At t3, the children of the intervention group had an average score of 8.35 (SD = 2.64), whereas the children of the control group had an average score of 6.27 (SD = 3.57). Children of the intervention group scored significantly higher at t3 [F(1, 37) = 9.23, p < 0.01, partial eta^2^ = .16]. For the Digit Span forward, children at or below the 50th percentile obtained a score of 5 points or less at t1. 41 children belonged to the intervention group and 30 to the control group. At t2, the children of the intervention group had an average score of 5.22 (SD = 1.55) and the children of the control group an average score of 4.45 (SD = 1.63), marking a significant difference [F(1, 51) = 4.27, p < 0.05, partial eta^2^ = .07]. Of the children scoring at or below the 50^th^ percentile on the Hearts & Flowers congruent (score of 24.5 or lower), 34 belonged to the intervention and 31 to the control group. The children of the intervention group obtained an average score of 24.90 (SD = 11.44) at t2 and a score of 28.48 (SD = 11.21) at t3. The children of the control group a score of 19.83 (SD = 10.50) at t2 and a score of 22.54 (SD = 10.33) at t3. A marginally significant difference were found for both t2 [F(1, 46) = 3.59, p = 0.06, partial eta^2^ = .07] and t3 [F(1, 45) = 2.96, p = 0.09, partial eta^2^ = .06].

We also assessed the teacher satisfaction regarding several aspects of the EMIL training. Mean levels of satisfaction for the five aspects (learning contents, instruction methods, relevance for teaching practice, learning outcomes, and collaboration with the instructors) were calculated across all participating teachers and training sessions. The majority of the teachers who participated in the EMIL training reported high satisfaction across all five aspects (mean rating score of 1.5 out of 6, SD = 0.70). None of the five questions had a mean satisfaction rating less than 1.8.

## Discussion

The aim of the present study was to evaluate the effects of an intensive teacher training on children’s EFs. The results showed that children whose teachers took part in the EMIL training program obtained significant higher scores on three out of seven EF measures (inhibitory control, visual-spatial working memory, and combined EFs) compared to a control group. Although significant gains were found, the number of effects as well as the effect sizes were, contrary to our expectations, quite small. This might be due to the fact that the intervention did not focus on children with EF deficits or lower socio-economic background. Instead, EMIL was applied with a middle-class sample and with all children visiting the preschool taking part in the intervention. Children from low-income families often display greater shortcoming in regard to their EFs and self-regulation skills [67, 68]. Other intervention studies found that only those children starting out with low EF scores show significant improvements but not those starting out high [38, 49, 66]. In our study post-hoc analyses for children scoring low at the first assessment revealed similar although not the same results as for the overall sample. Again, children of the intervention group scored significantly higher on inhibitory control at t2 and visual-spatial working memory at t3. Therefore, the differences found for the whole sample may be attributed to the sub-sample of children with low initial EF scores. As they started out lower, there was more room for improvement, which in turn led to greater variability in the post-test scores making it easier to identify changes. In addition to the results for the whole sample, a significant difference in phonological memory capacity was found in favor of the intervention group once only children with low EFs were taken into account. Also, the effect sizes were larger than the ones for the whole sample, although still quite small.

The preschools in our study were located in the same school district; hence, their classroom resources, staffing structure, and educational level were similar. The preschools were well-matched at school level and then randomly assigned to intervention and control group. ANCOVAs controlled for age, parental educational and occupational levels as well as score on pre-test. Therefore, children of the intervention group did not perform better because they were older, more economically advantaged, had more highly educated parents, or started out with higher scores than the control group as the analysis controlled for the influence of those factors. Unfortunately, the inclusion of several background variables in the analyses also resulted in a loss of power due to missing data.

There are several potential reasons that significant differences and near-significant trends only emerged for those three measures but not for others. One reason could be that the EMIL teachers supported the skills required in those three tests especially well with the activities they implemented and the way they guided the children. Teachers were not aware of the tests that were administered. Therefore, it can be dismissed that they trained exactly what the tests asked of the children. However, turn taking and collaboration, as assessed by the Tower test, makes up an important part of preschool life. Children in the intervention group may have received better support by their teachers when practicing collaborative play and sharing as a result of the EMIL training. It is assumed that EMIL influenced the guidance that teachers provided to the children of the intervention group as well as the implementation of fitting and well-considered activities thereby offering children more opportunities to develop their EFs. This in turn may have led to higher scores on the inhibitory control test. The gains in visual-spatial and phonological working memory could be attributed to the implementation of various activities to strengthen children’s working memory, e.g., challenging the children to get a number of items from the kitchen when setting the table. Also, preschool teachers reported changes in the way they communicated with the children, which might have taxed—among other things—children’s working memory. By returning questions to the children (‘You want to color. Okay! What materials do you need for water coloring and where do you find them?’) and involving them in their own thought processes (e.g., to find a solution or a compromise: ‘I wonder how we can solve this situation. Do you have an idea?’), children were encouraged to think for themselves. Learning how to scaffold children in their own thinking process (e.g., by given prompts or summarizing important facts) was an important part of the teacher training. However, it has to be noted, that the Digit Span backwards also measures phonological working memory but children showed no improvement in this test. Looking at the mean scores for this specific test, it becomes clear that it was challenging for a lot of children, as it showed clear floor effects.

It is difficult to explain the lack of results regarding the HTKS and the Day & Night. Both are rather ‘playful’ measures that resemble games that are assumed to require EFs such as ‘Simon says’. Taking a closer look at well-known games and discussing the load they may put on children’s EFs was part of the teacher training and have been implemented by the teachers of the intervention group. It may be though that those kinds of games are such a common activity in preschools that they were also played frequently with the children of the control group.

Some differences became apparent already at t2, but did not last and could not be detected at the third assessment whereas other effects emerged only at t3, three months after the teacher training had ended. It could be argued that the teacher training sped up the development of EFs in the children of the intervention group and that the children of the control group simply caught up six months later. It could also be the case that the fade-out of the intervention resulted in a drop of motivation among the teachers and thereby in a drop of EF activities.

Another reason for the low number of significant results may be that the intervention led to gains in children’s EFs but in ways not detected by the EF tests chosen for the evaluation [14]. The frequently described difficulty of choosing appropriate measures for preschoolers that assess EFs in reliable way may apply here [69].

During the training sessions, teachers often reported changes that they had perceived in their children’s everyday behavior. They had observed that children concentrated better on what they are doing, collaborated more, helped each other out, and solved problems more independently. It would have been useful to implement objective observation tools to track putative changes not only in children’s EFs but also in their self-regulation as its closer related to everyday activities [70].

The obtained results are in line with the extant body of research on preschool programs aiming to improve EFs in children. Evaluation studies of teacher trainings, e.g., Tools of the Mind [39] and Promoting Alternative Thinking Strategies (PATHS) [71], indicate that modifications in daily teaching practice can lead to small but significant gains in preschooler’s self-regulation skills and EFs. Curricula that practice diverse skills and all EFs all day long in preschool could produce widespread cognitive benefits [72]. An all-day Tools of the Mind curriculum for example showed greater benefits on children’s EFs than a Tools add-on to existing curricula [72]. However, it should be noted that some studies on the Tools of the Mind program have failed to find significant results [40]. Evaluations suggest that PATHS leads to higher emotional knowledge skills and children were rated as more socially competent than the children in the control group [73]. But here, too, the effects on EFs are rather small [38]. In general, most study results should be treated with caution: Some studies lack a control group or a baseline measure [10]. Control variables such as maternal education and socio-economic status, which are associated with children’s EFs [41–44], are often not accounted for. And when intervention effects are found, they are often over-interpreted [14].

Despite the small effect sizes and methodological issues, there are still some promising approaches on how to strengthen EF in preschool children. Then why was a new intervention developed? The contents and structure of the mostly US-American programs could not be simply implemented in Germany, because there are critical differences in the way that preschools work in the USA and Germany. More than 13% of German preschools have open classrooms where children can walk in and out [54]. Much of the time is dedicated to children’s free play. Furthermore, German preschools do not follow any predefined educational curricular for reading, writing, and arithmetic. These academic skills are taught in primary schools. Therefore, preschool teachers and their administrations have a lot of freedom to structure their day according to their needs and are hesitant to exchange this liberty for rigid programs. PATHS is a program with fixed content that requires a class structure. Tools of the Mind is more open and flexible but incorporates the enhancements of EF skills also in academic learning settings. Therefore, a new intervention program, EMIL, was developed to support the EFs of children in German preschools. The EMIL intervention, as described in the methods section, works solely with the preschool teachers who then implement changes to their classroom practice. The participating teams of preschool teachers developed ideas on how to adapt their pedagogical practices and everyday activities to promote their children’s EFs. As a result, they could respond more appropriately to the needs of their children and apply changes to areas that they thought needed attention (e.g., physical activities, pretend-play, interior design). As the teacher training involved sessions with the whole preschool teams, changes in time and room structure could be decided on right away and put to practice immediately thereafter. For a feasible and sustainable implementation with high fidelity, it was decided to train preschool teachers and not to use experts from outside [49]. The implementation in each individual preschool did not involve costly materials. In order to reach as many children as possible, it is important that the implementation of an intervention can be easily translated in every preschool setting and does not require expensive materials [49]. No special equipment is needed to address the four main aspects of EMIL (attitude, interaction & communication, structures, and activities). Once teachers learned about the connection between these aspects and the enhancement of EFs, they could identify simple strategies to support their children. Considering both aspects that EMIL trained the teachers of the preschools and that there is no additional equipment needed, we assume that the EMIL intervention is a very feasible way to support children’s EFs. This assumption is also supported by the results of the questionnaires which demonstrated the high satisfaction of the teachers with the EMIL training.

The translation of the teacher training into preschool practice by teachers depending on the circumstances in their preschools also created drawbacks for the study. Measures of treatment fidelity to track to what extent the intervention has been implemented by the teachers [40] were not implemented as preschools were encouraged to find their own translations. Each preschool worked on what they perceived needed most attention. The lack of standardization is a limitation of the study but is at the same time a feature and lies in the heart of the intervention idea. EMIL was designed to be readily implemented in German preschools as an ecologically valid intervention that continues after researchers have finished their work. The lack of standardization makes it harder to find substantial effects. But effects were found. Possibly they were found because of the flexibility and customizability of the EMIL practices. The involvement of the teachers in how to implement EMIL in their preschools was meant to make sure that EMIL practices were anchored into everyday activities in every classroom. Therefore, it can be assumed that children had more exposure to EF enhancing practices.

A second limitation of the study is that only a passive and no active control group was recruited. The teachers of the control group did not receive any standardized intervention. However, German preschool teachers are entitled to five days per year of continuing education. The control group received over the course of the intervention their individually chosen training courses provided by the school district. In this respect both groups differ little as both received teacher trainings. Still, it cannot be ruled out that teachers taking part in EMIL were more motivated through their participation as a team than the teachers in the control group.

The current study is limited by the relatively small sample size due to missing data. Larger samples that enable analyses to account for the nested structure of data as well as follow-ups to assess longer-term effects of training at 6 months and beyond are needed to more rigorously assess the effects and the longevity of training. Assessment would be augmented by incorporating other objective measures that are appropriate for children between three and six years of age. It is assumed that EMIL influenced the teaching practice and the guidance that teachers provide to the children. However, as project fidelity has not been observed in an objective manner, it is difficult to put a finger on the active ingredients. For future studies it would be useful to track which activities have been implemented by the preschool teachers in order to gain some insight on how much of the content of the teacher training was transferred into everyday practice. Additionally, it should be considered to implement recurring check-ins with the teacher teams or to send out monthly newsletters with a short theoretical input and ideas for pedagogical activities to hold up motivation after the fade-out. Also, preschool teachers showed high levels of satisfaction with the training contents and reported changes in children’s everyday behavior but again, these were not yet measured in an objective manner outside of the standardized EFs tests. Still, the EMIL teacher training program led presumably to EMIL practices in preschools that enhanced EFs in children in return. The findings indicate that teacher training could not only be an ecologically valid intervention but also lead to significant benefits for children’s EFs. But although the approach appears promising, larger randomized-controlled trials are needed to determine how children can be effectively supported in their development of EFs.

## Conclusion

EMIL is a preschool intervention program that aims to enhance EFs by considering the individual needs of the children, the teachers, and structures of the preschool. It can be customized for every preschool. The obtained results add to the evidence that training of preschool teachers may lead to significant gains in children’s EFs and especially in those starting out low. Given the centrality of EFs to school readiness, social-emotional competences, and academic achievement, it is of great interest to equip teachers with the knowledge and the tools necessary to support children in this regard early in life.
